# Update on the Role of Autologous Hematopoietic Stem Cell Transplantation in Multiple Myeloma

**DOI:** 10.4084/MJHID.2012.069

**Published:** 2012-11-05

**Authors:** P. Tosi, M. Imola, A. M. Mianulli, S. Tomassetti, A. Merli, A. Molinari, S. Mangianti, M. Ratta, A. Isidori, G. Visani

**Affiliations:** 1 Hematology Unit, Department of Oncology and Hematology, Infermi Hospital Rimini Italy; 2 Hematology and Transplant Center AORMN Marche Nord Pesaro Italy

## Abstract

Autologous stem cell transplantation is considered the standard of care for multiple myeloma patients aged < 65 years with no relevant comorbidities. The addition of drugs acting both on bone marrow microenvironment and on neoplastic plasma cells has significantly increased the proportion of patients achieving a complete remission after induction therapy, and these results are mantained after high-dose melphalan, leading to a prolonged disease control. Studies are being carried out in order to evaluate whether short term consolidation or long-term maintenance therapy can result into disease eradication at the molecular level thus increasing also patients survival. The efficacy of these new drugs has raised the issue of deferring the transplant after achiving a second response upon relapse. Another controversial point is the optimal treatment strategy for high-risk patients, that do not benefit from autologous stem cell transplantation and for whom the efficacy of new drugs is still matter of debate.

## Introduction and Hystorical Background

Multiple myeloma (MM) is a clonal B cell disorder characterized by proliferation and accumulation of B lymphocytes and plasma cells in the bone marrow and,and, more rarely, at extramedullary sites. Its annual incidence is 6/100000 in western countries, thus representing the second most common hematological malignancy after non Hodgkin lymphomas.^1^

For many years the combination of melphalan and prednisone (MP), that was developed in the early sixties by Bergsagel et al,^2^ has been considered the gold standard treatment for MM, as different polychemotherapy regimens failed to demonstrate a better efficacy.^3^ MP was able to induce a response in over 40% of treated patients; complete responses, however, were achieved in less than 5% of the cases, and overall patients survival did not exceeded 3 years. The first step towards introduction of autologous stem cell transplantation in MM was represented by in vitro studies showing a a dose-response effect of melphalan in MM cells.^4^ The potential to overcome resistance to melphalan by using higher doses of the drug was subsequently explored in vivo;^5^ 27% previously untreated patients reached a complete response (CR), and this translated into a prolonged survival, even though treatment related mortality was unacceptably high. In order to reduce the duration of profound cytopenia related to the use of high dose melphalan (HDM), autologous stem cell rescue was then introduced in the clinical practice, initially for relapsed/refractory disease, then for newly diagnosed MM.^6^,^7^ The formal demonstration that autologous stem cell transplantation (ASCT) is superior to conventional chemotherapy in terms of response, duration of response and survival, came from two randomized trials, the first one from the Intergroup Francophone du Myeloma (IFM)^8^ and the second one from the Medical Research Council (MRC).^9^ In order to ameliorate these results, the application of two subsequent ASCTs was then explored by IFM^10^ and by the Bologna group;^11^ both studies demonstrated an improvement in response rate and event-free survival (EFS); however only the French study was able to show a survival advantage for patients receiving a double ASCT. Further analysis of the IFM trial showed that a second ASCT could result into an increased OS only in patients failing to achieve at least a very good partial response (VGPR)^10^ after the first ASCT, these data were in agreement with a subanalysis of the Bologna trial showing an improved event-free survival (EFS) after a second ASCT in patients failing to achieve at least a near-CR after the first one.^11^

While the use of a double ASCT is still matter of debate, from late nineties on, a single ASCT has been referred as the standard of care for newly diagnosed MM patients aged <60–65 years with no relevant comorbidities, this in accord with the upper age limit that has been considered appropriate for patients with other kinds of hematological malignancies, even though interesting results were obtained also in older patient populations.^12^

## The Role of CR

When MP was the only available therapeutic strategy for MM, the attainment of CR was no matter of concern as only a minority of patients could achieve a minimal residual disease status. The introducion of more aggressive therapeutic programs including ASCT, prompted a better evaluation of minimal residual disease, including also cytofluorimetric^13^ and molecular techniques.^14^ At present, the International Myeloma Working Group (IMWG) has provided the definition of "stringent CR" including negative serum/urine immunofixation together with a normal serum free-light chain ratio and absence of clonal plasma cells in the bone marrow.^15^ Several groups have analyzed the relationship beween CR and patients outcome, and have pointed out that CR is a strong predictor of survival,^16^ expecially when extended over several years;^17^ for this reason it is now generally recognized that every effort should be made in order to achieve maximal disease eradication through the various phases of the treatment program.^18^

## Incorporation of Novel Drugs in Induction Phase

In addition to the clinical benefit offered by ASCT, in recent years the therapeutic results for MM have significantly improved due to the availability of drugs that are active both on neoplastic plasma cells and on bone marrow microenvironment, such as thalidomide, lenalidomide and bortezomib. After testing in patients with advanced, relapsed/refractory disease, these compounds were evaluated in clinical trials in the framework of induction therapy prior to ASCT in newly diagnosed patients in order to increase the depth of response thus improving patients outcome. Thalidomide was the first agent included in induction therapy for newly diagnosed MM patients eligible for ASCT; the drug was used in combination to high-dose dexamethasone (TD) (Table 1) yielding interesting results as compared to conventional chemotherapy in a case-match retrospective analysis^19^ or to high-dose dexamethasone in a prospective randomized trial.^20^ In a further randomized trial (Total Therapy 2) thalidomide was continuously applied in the various phases of the whole treatment program until patient relapse,^21^ again an advantage in terms of CR rate and EFS was observed in patients treated with thalidomide as compared to those not receiving the drug, but OS was similar in the two groups of patients. Subsequent trials were designed aiming at evaluating the combination of TD with doxorubicin;^22^ a significant improvement in response rate was observed as compared to conventional chemotherapy (VAD) (Table 1). Bortezomib was tested in combination to dexamethasone (VD) in a phase II study;^23^ a VGPR rate of over 30% was achieved after induction and upgraded to over 50% after ASCT (Table 2). A further phase II study was designed aiming at comparing VD to conventional vincristin-doxorubicin-dexmethasone (VAD);^24^ again the arm treated with the novel regimen showed a significantly higher response rate (38% VGPR or better vs 15%) that was confirmed after ASCT. The combination of VD with cyclophosphamide (VCD) was able to induce a VGPR or better in over 60% of the patients,^25^ similar results were reported using VD+ doxorubicin (PAD).^26^ Lenalidomide was studied in a randomized trial in combination to high (RD) vs low (Rd) doses dexamethasone,^27^ after 4 courses patients were allowed to undergo ASCT or to proceed with the same therapy; even though response rate was significantly higher in the RD group, survival was the same due to the higher toxicity experienced by the RD group.

A further improvement in the results obtained with novel drugs±steroids±chemotherapy was achieved combining two novel drugs with dexamethasone (Table 2). The combination bortezomib-thalidomide and dexamethasone (VTD) was randomly compared to thalidomide-dexamethasone (TD) as induction therapy prior to ASCT, yielding a significant advantage in terms of response, both CR and VGPR.^28^ These data were confirmed by a recent study of the Pethema group.^29^ A bortezomib+thalidomide-containing regimen was also used in Total Therapy 3 trial,^30^ in the context of a polychemotherapy program involving induction, ASCT, consolidation and maintenance; as compared to Total Therapy 2, in which only TD was used,^21^ a significant prolongation of EFS was observed. These results so far indicate that induction therapy in preparation to ASCT should include bortezomib+dexamethasone + an immunomodulating agent, either thalidomide or lenalidomide, that is presently being explored in phase II trials.^31^

## Controversial Issues

### Consolidation, Maintenance or Both?

The administration of some kind of treatment upon completion of major therapy in order to improve/maintain its efficacy represents the standard of care in several lymphoproliferative neoplasms such as acute lymphoblastic leukemia, low grade lymphoma or mantle cell lymphoma, and for this reason it has been considered an attractive option also for MM. Several groups have addressed the issue of post transplantation treatment, and interesting results have been reported; at present, however, no data can definitely support a treatment over another, and no drug has been formally approved for the therapy of MM at this disease stage. Consolidation therapy is defined as a short course of treatment administered after ASCT aiming at further reduce tumor load (Table 3). A study from the nordic group^32^ has evaluated the efficacy of a short course of Bortezomib, and an increased percentage of CRs was observed. Two different studies analyzed the effects of a short course of Bortezomib-thalidomide-dexamethasone (VTD) administered as consolidation after ASCT, both trials showed that a molecular response can be achieved in up to 60% of the patients.^33^–^35^ Maintenance therapy is defined as long-term treatment aiming at preventing disease recurrence or progression. Alpha interferon has been widely tested after ASCT and despite two reports showing an improved survival, side effects greatly overcome the possible advantage, so that this approach has been definitely abandoned.^36^ A limited efficacy was also reported with long term use of steroids.^37^ Thalidomide has been studied in six trials^21^,^22^,^38^–^41^ (Table 4), in 3 of which the drug was used also in induction phase. Although all the trials showed an advantage in terms of EFS or PFS; an OS advantage for patients treated with thalidomide was observed only in 2 trials. A major concern regarding the use of this drug as maintenance therapy is the high percentage of patients dropping out due to long term side effects, specifically peripheral neuropathy.^38^–^41^ Furthermore, the likelihood of selecting MM clones resistant to thalidomide and responsible for short post-relapse survival should probably be taken into consideration^21^–^22^,^41^ as well as the limited efficacy of the drugs in case of poor-risk cytogenetic.^41^ Due to its favorable toxicity profile, and specifically to the lack of long-term neurological toxicity, lenalidomide has been tested as maintenance therapy in two randomized studies,^42^–^43^ both of them showed a significant advance in TTP, while OS was significantly improved only in one study.^43^ Side effects were mainly hematological, a higher percentage of second primary malignancies were observed in Lenalidomide-treated patients,^42^,^43^ however this data need further observation as it is clear that survival benefit outgrows the risk of death from second malignancies.^44^ A recent report analyzed the role of bortezomib maintenance after ASCT;^26^ patients showed a significant advantage in terms of PFS and OS, even though the potential of neurological toxicity should be taken into consideration.

Despite these interesting results, however, data are not mature to recommend a specific strategy, and the issue of consolidation and/or maintenance treatment remains still debated

### Upfront or Salvage ASCT?

Early studies on ASCT in MM were performed in patients with relapsed/refractory disease but, due to the poor result that were obtained,^45^ the procedure is now preferentially employed in newly diagnosed patients.^46^ Furthermore, a timely-dependant application of ASCT seems to be crucial in determining an optimal response.^47^ A randomized study from the French group,^48^ however, demonstrated a comparable outcome in terms of survival in patients undergoing early vs deferred ASCT (64.4 vs 64 months OS). These data were obtained when only chemotherapeutic agents were available; it is now evident that new drugs, when applied during induction, are able to determine a deeper response than that obtained with conventional chemotherapy combinations. Several groups have thus designed studies aimed at evaluating efficacy of long term treatment with new drugs as compared to ASCT,^49^,^50^ applying transplant only upon relapse. Results that have been published so far failed to show a difference in patients survival even though early ASCT is related to a shorter duration of treatment and drug exposure. A recent retrospective study has shown that, in patients treated with thalidomide or lenalidomide followed by early stem cell mobilization,^51^ comparable results were achieved after early vs late ASCT. Data from further studies are awaited

### Is ASCT Feasible in Elderly Patients?

Patients aged > 65 years are not considered good candidates to ASCT as their survival is significantly shorter than that observed in younger patients (50% vs 68% at 5 years, 52). Several reports, however, have identified a “grey zone” represented by patients aged 65–70 in good clinical conditions, that could potentially take advantage from this procedure. In particular, a randomized study conducted in these patients has demonstrated that intermediate dose melphalan (10mg/sqm) with PBSC support results into a significantly prolonged event-free and overall survival as compared to melphalan-prednisone.^53^ On the other hand, a later study conducted in older patients (65–75 years) failed to show an advantage of intermediate dose melphalan as compared to MP, and both regimens were inferior to the combination melphalan-prednisone and thalidomide.^54^ At present, however, no data can unequivocably establish whether an ASCT program including new drugs can be useful in older patients as it happens in younger ones. At present only one phase II study has been reported, aimed at evaluating the toxicity and the efficacy of bortezomib and lenalidomide included in pre-transplant induction and post transplant consolidation and maintenance in patients aged 65–75 years.^55^ The percentage of patients obtaining a CR increased progressively through the various phases of the treatment program (13% after induction, 43% after transplant and 73% during consolidation/maintenance) and hematological and non-hematological toxicities were acceptable. These data indicate that, in selected elderly patients, an ASCT program including new drugs can be safely performed, thus representing a possible therapeutic option.

### Is ASCT the Best Treatment for High-Risk Patients?

In recent years, many attempts have been made in order to identify patients at high risk of relapse and poor survival, and several parameters have been taken into consideration. The simplest and cheapest one is the International Staging System (ISS) prognostic model,^56^ designed by the IMWG, based on beta-2 microglobulin and albumin level; a significantly different survival (62 months, 44 months and 29 months) was shown in stage 1, 2 or 3 patients, respectively. The major pitfall of this risk stratification is that it does not take into account cytogenetic alterations, that are now considered the main parameter affecting patients prognosis. No agreement does still exist on which, among fluorescence-in situ hybridization (FISH), comparative genomic hybridization (CGH) and gene expression profile (GEP) is the best method to use in order to detect chromosomal abnormalities. However, patients showing t (4;14), t (14;16) deletion 17q (57) or 1q abnormalities^57^,^58^ carry a worse prognosis and should be treated differently from patients with no chromosomal abnormality.^59^ Very few data however, are presently available concerning the efficacy of different therapeutic regimens in poor risk patients. A bortezomib -containing induction therapy seems to be able to overcome the adverse prognosis carried by t(4;14).^28^ This is not the case for thalidomide,^60^ especially in maintenance trials^37^ while conflicting results were reported regarding lenalidomide-dexamethasone induction.^61^ On the other hand, patients with 17q deletion seem not to benefit from Bortezomib followed by ASCT.^62^ Dose dense regimens, upfront myeloablative allogeneic stem cell transplantation or novel agents are presently proposed for high risk patients, in the context of clinical trials, aiming at finding a proper therapeutic approach.

### Autologous, Allogeneic or Tandem Autologous-Allogeneic SCT?

Myeloablative allogeneic bone marrow transplantation (allo-BMT) or, later on, allo-SCT for the treatment of MM was introduced in the early 80s by several Institutions.^63^ This procedure allowed to demonstrate that high dose chemo/radiotherapy coupled with the graft versus myeloma (GVM) effect could overcome drug resistance and induce long-lasting complete remission; transplant-related mortality (TRM), however, remained a major issue for many years, with most of the trials reporting mortality rates ranging from 30 to 50%.^64^–^65^ On the other hand, allo-SCT can result into a more frequent molecular CR and decreased probability of relapse as compared to ASCT;^66^ therefore it is likely that this procedure is probably the only therapeutic approach which has the potential ability to eradicate the myeloma clone. A decrease in TRM could be achieved using of non-myeloablative preparative regimens (RIC-allo-SCT), aimed at reducing conditioning-related toxicity while sparing GVM effect. A great variety of preparative regimens have been used, either including low dose (2Gy) total body irradiation with fludarabine or intermediate-dose melphalan plus fludarabine; a favorable outcome is more frequently observed in non-heavily pretreated patients and in chemosensitive disease.^67^ A tandem strategy of high-dose melphalan and ASCT followed by RIC-allo-SCT has been propsed by several groups, in order to further decrease tumor burden prior to induce GVM effect. A direct comparison of double ASCT versus tandem ASCT followed by RIC-allo-SCT led to controversial results; with the autologous+allogeneic strategy resulting superior according to Bruno and Bjorkstrand^68^–^69^ and inferior according to Moreau and Krishnan.^70^–^71^ A recently published meta analysis concluded that ASCT followed by RIC-allo-SCT is associated with a higher percentage of CR, but TRM is also higher, thus leading to lack of improvement of PFS and OS.^72^

#### Concluding Remarks

In the last few years the outcome of MM patients has significantly improved with the introduction of novel drugs in the clinical practice. The inclusion of thalidomide, lenalidomide or bortezomib in various combinations, in the different phases of an ASCT program, increases the percentage of patients achieving a CR, thus potentially leading to patients cure. Data are not mature, so far, to establish whether a combination of new drugs, administered for a prolonged period of time, could render ASCT unnecessary. At present, in many US Institutions, both physicians and patients are in favor of a delayed ASCT policy, in order to avoid complications related to the period of myelosuppression related to the procedure. It cannot be taken for granted, however, that patients quality of life is worse in case of a short time myelosuppression as in ASCT, rather than in case ofa prolonged therapy with any of the new drugs that are presently available and whose side effects are well known. At present, at least in Europe, ASCT is still considered the standard of care for young patients with newly diagnosed MM, and the issue is how the results can be further improved. A number of new drugs are presently being tested in MM, at various disease phases. Among them carfilzomib, an irreversible proteasome inhibitor, that after having proven effective in relapsed/refractory disease, has been tested in combination with lenalidomide in newly diagnosed MM patients^73^ inducing up to 40% stringently defined CR. Pomalidomide, a thalidomide derivative, has demonstrated to be effective even in lenalidomide or bortazomib-refractory patients.^74^ These drugs will be probably included into induction therapy prior to ASCT in order to further improve disease eradication.

## Figures and Tables

**Table 1:** Thalidomide-containing induction regimens

		Induction	Post ASCT		
Author (reference)	Regimen	≥VGPR (%)	≥VGPR (%)	PFS	OS
Cavo (19)	TD	19	68	51% @4yrs	69% @5 yrs
Rajkumar (20)	TD	35	44	NR	NR
Lokhorst (22)	TAD	37	54	Median 34mos	Median 73 mos
Barlogie (21)	TT2	NR	62 (CR)	56% @5 yrs	65%@ 5 yrs

TD = thalidomide-dexamethasone; TAD = thalidomide-doxorubicin, dexamethasone; TT2= total therapy 2; VGPR = very-good partial remission; CR = Complete remission; NR = not reported, PFS = progression-free survival, OS = overall survival

**Table 2:** Major drug combinations used as induction therapy

		Induction	Post ASCT		
Author (reference)	Regimen	≥VGPR (%)	≥VGPR (%)	PFS	OS
Harousseau (23)	VD	38	54	36 mos	81%@ 3 yrs
Cavo (28)	VTD	62	82	68% @ 3 yrs	86% @ 3 yrs
Sonneveld (26)	PAD	42	61	35 mos	NR
Reeder (25)	VCD	61	74	NR	NR
Rajkumar (27)	Rd	40	NR	63% @ 2 yrs	92% @ 3 yrs
Rosinol (29)	VTD	60	46 (CR)	56.2 mos	74% @ 4 years
Richardson (31)	RVD	61	NR	75% @ 18 mos	97% @ 18 mos

VD = bortezomib-dexamethasone; VTD= bortezomib-thalidomide-dexamethasone, PAD=Bortezomib-doxorubicin, dexamethasone ; VCD = bortezomib-cyclophosphamide-dexamethasone; Rd = lenalidomide-low dose dexamethasone; RVD = lenalidomide-bortezomib-dexamethasone; VGPR = very-good partial remission; NR = not reported, PFS = progression-free survival, OS = overall survival

**Table 3:** Regimens used as consolidation therapy

Author (reference)	Regimen	Nr of courses	CR (%)
Mellqvist (32)	V	6	45 (near-CR)
Ladetto (33)	VTD	4	49 (CR with negative immunofixation)
Cavo (34)	VTD	2	61 (CR with negative immunofixation)

V = bortezomib; VTD = bortezomib-thalidomide-dexamethasone; CR = Complete remission

**Table 4:** Maintenance regimens

Author	Regimen	Duration	PFS	Longer OS compared to control
Spencer (39)	Thalidomide/prednisone	12 mos	42% @ 3 yrs	yes
Attal (38)	Thalidomide/Pamidronate	Until PD	37% @ 5 yrs	no
Barlogie (21)	Thalidomide	Until PD	57% @ 5 yrs	no
Lokhorst (22)	Thalidomide	Until PD	Median 34 mos	no
Morgan (41)	Thalidomide	Until PD	Median 23 mos	no
Stewart (40)	Thalidomide	Until PD	Median 28 mos	no
Attal (42)	Lenalidomide	Until PD	Median 41 mos	no
McCarthy (43)	Lenalidomide	Until PD	Median 48 mos	yes
Sonneveld (26)	Bortezomib	2 yrs	Median 36 mos	yes

PD = progressive disease; PFS = progression-free survival; OS = overall survival
